# Bioaugmentation potential of inoculum derived from anaerobic digestion feedstock for enhanced methane production using water hyacinth

**DOI:** 10.1007/s11274-023-03600-9

**Published:** 2023-04-10

**Authors:** Linda U. Obi, Ashira Roopnarain, Memory Tekere, Rasheed A. Adeleke

**Affiliations:** 1 Department of Environmental Sciences, University of South Africa, Johannesburg, South Africa; 2 Microbiology and Environmental Biotechnology Research Group, Institute for Soil, Climate and Water, Agricultural Research Council, Arcadia, Pretoria, 0083 South Africa; 3 Unit for Environment Science and Management, North-West University (Potchefstroom Campus), Potchefstroom, South Africa; 4 Department of Biological Sciences, Godfrey Okoye University, Jideofor St, Thinkers Corner, Enugu, 400001 Enugu State Nigeria

**Keywords:** Anaerobic digestion, Biogas, Digestate, Methane, Soil ameliorant, Water hyacinth

## Abstract

The utilisation of water hyacinth for production of biogas is considered to be a solution to both its control and the global renewable energy challenge. In this instance, an investigation was conducted to evaluate the potential of water hyacinth inoculum to enhance methane production during anaerobic digestion (AD). Chopped whole water hyacinth (10% (w/v)) was digested to prepare an inoculum consisting mainly of water hyacinth indigenous microbes. The inoculum was incorporated in the AD of freshly chopped whole water hyacinth to set up different ratios of water hyacinth inoculum and water hyacinth mixture with appropriate controls. The results of batch tests with water hyacinth inoculum showed a maximal cumulative volume of 211.67 ml of methane after 29 days of AD as opposed to 88.6 ml of methane generated from the control treatment without inoculum. In addition to improving methane production, inclusion of water hyacinth inoculum reduced the electrical conductivity (EC) values of the resultant digestate, and, amplification of *nifH* and *phoD* genes in the digestate accentuates it as a potential soil ameliorant. This study provides an insight into the potential of water hyacinth inoculum to enhance methane production and contribute to the feasibility of the digestate as a soil fertility enhancer.

**Supplementary Information:**

The online version contains supplementary material available at 10.1007/s11274-023-03600-9.

## Introduction

Recent studies have been conducted on the efficiency of lignocellulosic substrates in the production of biogas (Martínez-Gutiérrez 2018; Ferraro et al. 2020; Kumar et al. 2020). Such substrates include bamboo wastes (Shen et al. 2014), oil palm mesocarp (Saidu et al. 2014), olive wastes and citrus pulp (Panuccio et al. 2016), corn stover (Schroyen et al. 2014), napier grass (Lianhua et al. 2014) and water hyacinth (Lin et al. 2015; Nkuna et al. 2019), amongst others. These substrates were selected for anaerobic digestion (AD) to produce biogas based on their chemical composition, wide availability and the challenges they pose on the environment (Shenoy et al. 2022). In addition to the aforementioned qualities, low lignin content of water hyacinth and its possession of plants’ essential nutrients make it a suitable substrate for efficient production of biogas and soil ameliorant (Njogu et al. 2021; Barua and Kalamdhad 2019). The use of water hyacinth to generate biogas is an eco-friendly and innovative means of managing this intrusive aquatic weed (Roopnarain et al. 2019). Water hyacinth is a menace in the aquatic environments as its rapid proliferation hinders many biological and socioeconomic activities where they are resident (Honlah et al. 2019). These activities range from the reduction in the level of dissolved oxygen in the water, hindrance of photosynthetic activities of submerged plants to obstruction of recreational/economic activities in the aquatic environments (Njogu et al. 2021). Besides its wide availability due to its high proliferation capability, water hyacinth’s elevated cellulose and hemicellulose content as well as low lignin composition contributes to its prospect as a substrate for the production of biogas (Sindhu et al. 2017). However, the inaccessibility of the lignocellulosic portion of water hyacinth to microorganisms makes its biodegradation a challenge (Sarto et al. 2019). To enhance the bioavailability of the lignocellulosic portion of the hyacinth and improve enzymatic hydrolysis (microbial degradability), additional steps such as pretreatment and inoculum addition are often required during the AD process for increased biogas production (Achinas et al. 2019). Nevertheless, the ecological sustainability of some pretreatment techniques on lignocellulosic materials is still a challenge. Pretreatment methods that involve high pressure and heat application as well as the use of oxidizing agents lead to high energy consumption, and accumulation of salts in the digesters which could negatively impact methanogenic activities (Yu et al. 2018; Chen et al. 2020). In addition to microbial community stability, incorporation of inoculum during the metabolic process of AD is an efficient technique as the inoculum enhances the degradation of substrate through improved enzyme activities (Dennis 2015). Several studies have been conducted on the relevance of water hyacinth for the production of biogas (Etta et al. 2017; Barua et al. 2019; Kunatsa et al. 2020; Unpaprom et al. 2021). However, the use of water hyacinth as a potential inoculum to enhance biogas production is yet to be investigated. A study that reported that biogas-producing microorganisms are innately linked to water hyacinth harvested from the Hartbeespoort dam in South Africa motivated the investigation into the potential of these microorganisms as an inoculum (Roopnarain et al. 2019).

Another significant part of AD of water hyacinth is the digestate. The digestate from anaerobic digesters is an environmentally friendly semi-liquid by-product which contains some plant growth promoting macro and micronutrients suggesting the prospective of the digestate to serve as a soil ameliorant (Sindhu et al. 2017). The digestate also contains live cells of different plant growth promoting microbial strains that could assist in improving plant health (Barua and Kalamdhad 2019; Risberg et al. 2017). Microorganisms resident in anaerobic digesters have been associated with the promotion of plant development and growth through siderophores and phytohormone production, solubilization of insoluble phosphate, zinc and potassium as well as fixing of atmospheric nitrogen (Souza et al. 2015; Khan et al. 2016). These microbes known as plant growth promoting microorganisms (PGPM) are capable of improving nutrient acquisition as well as metabolism and physiological processes in plants thus enhancing plant productivity (Liu et al. 2010; Adeleke et al. 2019). From the aforementioned reasons, soil ameliorants are promising alternatives to chemical fertilizers, which are associated with environmental pollution (Mukhuba et al. 2018). Studies have also portrayed the beneficial effect of the resultant effluent from the AD of water hyacinth as a soil ameliorant (Arutselvy et al. 2021; Ramirez et al. 2021; Unpaprom et al. 2021). However, digestate from the AD of water hyacinth that are potential soil ameliorants may contain trace amounts of heavy metals and other salts. This may occur as a result of water hyacinth being able to absorb and accumulate some other organic pollutants including heavy metals from polluted aquatic environment where they thrive (Mudhoo and Kumar 2013; Jones et al. 2018). The Hartbeespoort Dam based in South Africa is an example of a polluted aquatic ecosystem where extensive proliferation of water hyacinth is a problem. The dam is hypertrophic due to the discharge of agricultural, domestic, and industrial effluents (Atta et al. 2020). Water hyacinth, a known phytoremediation agent due to its high absorptive capacity, could contribute to the electrical conductivity (EC) values of digestate from AD of water hyacinth (Safauldeen et al. 2019; Peng et al. 2020). Decomposition of organic matter such as water hyacinth could potentially increase the salts and ions in the resultant effluent (Carmo et al. 2016). Application of such digestate as soil ameliorant could result in high EC values of soil leading to low crop productivity as EC is an indicator of soil health (Husson et al. 2018). Nevertheless, plants require some of these heavy metals at acceptable levels/concentrations for growth and productivity (Romero-Güiza et al. 2016). In addition, the presence of PGPM in the soil ameliorant has been known to alleviate the harmful effects of heavy metals on plants (Hassan et al. 2017). This study aims to ascertain the potential of inoculum derived from AD of water hyacinth to enhance methane production as well as the ideal mixing ratio of pre-treated water hyacinth and water hyacinth inoculum for optimal methane production. The study also hypothesizes the potential of digestate from AD of water hyacinth from the Hartbeespoort dam as soil ameliorant.

## Materials and methods

### Sampling

Permission to collect and utilize water hyacinth was granted by the Department of Environmental Affairs, South Africa (permit numbers 5086577918 and 5086577921). Water hyacinth (substrate) was wholly harvested from the Hartbeespoort dam that is situated in Madibeng district of the North West province of South Africa (25° 44ʹ 51ʺ S 27° 52ʹ 1ʺ E). The substrate which includes the leaves, stems and roots was transported in sterile storage containers to the Biogas laboratory at the Agricultural Research Council—Soil, Climate and Water, Pretoria, South Africa where it was pre-treated by cutting into small sizes of 2 cm × 2 cm prior to analysis.

### Experimental set-up

The substrate whole water hyacinth was characterized for physico-chemical properties which include dry matter, heavy metals, phosphorus, potassium, ammonium content, total solids (TS), volatile solids (VS), ash and pH using standard methods (APHA 2017). Concentrated acid digestion method (CADM) with Inductively Coupled Plasma Mass Spectrometry (ICP-MS) was used to quantify heavy metals concentration (Mukhuba et al. 2018). Bray 1 method was employed in extracting available phosphorus which was further analysed using a spectrophotometer (Mukhongo et al. 2017). The total solids (TS) and volatile solids (VS) content of the plant evaluated by oven drying samples at 105 °C for 24 h and combustion of dried samples at 550 °C for 6 h in a muffle furnace respectively (APHA 2017). Additional compositional analysis of the substrate include cellulose, hemicellulose, and lignin analyses of the substrate which was based on the Neutral Detergent Fiber (NDF), Acid Detergent Fiber (ADF), and Acid Detergent Lignin (ADL) composition of freeze-dried substrates (Van Soest et al. 1991; Hindrichsen et al. 2006).

Water hyacinth inoculum (whinc) was generated by AD of 10% (w/v) of freshly chopped whole water hyacinth (Wh) under rotatory incubation at 30 °C and 120 revolutions per minute (rpm) for 2 weeks. Freshly chopped Wh was mixed with the whinc in various ratios in 500 ml Schott glass bottles equipped with screw caps containing septa. All the mixing ratios including the quantity of Wh and whinc as shown on Table 1 had the same TS (2%). The volume of each of the treatments was bulked to 250 ml with tap water.

**Table 1:** Experimental design of the batch culture for biogas production from bioaugmentated water hyacinth

Treatments nos.	Ratio of Wh:whinc	Quantity of Wh (g)	Quantity of whinc (ml)
1	Wh:whinc 1:1	50	50
2	Wh:whinc 1:2	33.35	66.65
3	Wh:whinc 1:4	20	80
4	Wh:whinc 4:1	80	20
5	Wh:whinc 2:1	66.65	33.35
6	Wh:whinc 1:0 (control)	–	100
7	Wh:whinc 0:1 (control)	100	–

Water hyacinth was also digested without added water or water hyacinth inoculum in a separate treatment. This treatment was set up to evaluate the dry digestion of water hyacinth since the plant is constituted primarily of water. The nutrient and heavy metal composition of this treatment was not assessed due to significant reduction in the quantity of digestate. None of the treatments were purged with nitrogen gas prior to digestion. Treatments were set up in triplicate with appropriate controls. These treatments were digested as batch cultures until reduced CH_4_ and CO_2_ production was observed due to substrate depletion (29 days). The cultures were kept at 30 °C and 120 rpm (revolutions per minute) and biomethane production was monitored by means of Gas chromatography (GC) (SR1 8610C, CHROMPEC, Canada). The gas chromatograph was fitted with a thermal conductivity detector (TCD) and HayeSep D packed column for the analysis. With reference flow of 20 ml per minute and make up flow of Helium carrier gas at 10 ml/min, Temperature of the TCD was set at 155 °C. Initial oven temperature was set at 50 °C and held for 4 min, initial ramp temperature of 20 °C and final temperature of 220 °C. Two milliliter aliquots of gas was sampled from the headspace of the batch culture bottles by means of a gas tight syringe with Luer lock valve (SGE 10MDR-VLLMA-GT). The aliquot was injected into the GC for analysis of biogas composition (CH_4_ and CO_2_) at 3 day intervals. After 29 days of AD, the digestate produced was analysed for heavy metals and pH using standard methods for the examination of Water and Wastewater (APHA 2017) and a pH meter (Adwa AD1030) respectively.

The feedstock and digestate from different mixing ratios were characterised for heavy metals, phosphorus, potassium and ammonium content using the previously mentioned methods above. All physico-chemical analyses were done by the analytical laboratory of the Agricultural Research Council-Soil, Climate and Water, Pretoria, and Agricultural Research Council—Animal Production, Irene, South Africa.

### Microbial analysis

#### Identification of plant growth promoting genes

One millilitre of homogenised sample of the digestate was centrifuged at 10,000×*g* for 5 min to concentrate the sample. Genomic DNA was isolated from the pellet using the DNeasy PowerSoil extraction kit according to manufacturer’s protocol (Adeleke et al. 2010). Quantification of isolated DNA was executed with Qubit 2.0 Fluorometer (Invitrogen, Life Technologies, South Africa) and DNA extracts were stored at a temperature of −20 °C for further downstream applications (Roopnarain et al. 2017). The ability of the digestate to promote plant growth was ascertained by targeting the *nifH* gene for nitrogen fixation and the *phoD* gene for phosphate solubilisation using Polymerase Chain Reaction (PCR). The *nifH* gene was targeted with specific primers PolF (5′-TGC GAY CCS AAR GCB GAC TC-3′) and PolR (5′-ATS GCC ATC ATY TCR CCG GA-3′) (Qin et al. 2014; Niu et al. 2018). The *phoD* gene was targeted with ALPS-F730 (5′ CAG TGG GAC GAC CAC GAG GT-3′) and ALPS-R1101 (5′-GAG GCC GAT CGG CAT GTC G-3′) primers (Sakurai et al. 2008; Fraser et al. 2017). Amplification reaction mix of 25 µl was prepared and it consisted of 12.5 µl of One Taq 2 × Master Mix with standard buffer, 0.5 µl (10 µM) of each of the primers, 3 µl of DNA template and 8.5 µl of sterile distilled water. The reaction mix was preheated to 94 °C for 30 s in a BIORAD T100™ Thermal Cycle. Thirty cycles were run at 94 °C, 30 s; 55 °C, 1 min; 68 °C, 1 min and elongation followed at 68 °C for 5 min. The same amplification and cycling conditions were used for the amplification of the *phoD* genes but the annealing temperature was set at 57 °C for 1 min. Amplicon sizes and quality were verified by agarose gel electrophoresis and amplicons were preserved at −20 °C ( Obi et al. 2020).

#### Identification of bacterial isolates

Bacterial isolates obtained from the water hyacinth inoculum through cultivation on nutrient agar at 30 °C for 24 h were identified based on the partial sequence of 16S rRNA gene via colony polymerase chain reaction (colony PCR) with universal bacterial primer set, 27F and 1492R (annealing temperature = 53 °C for 1 min) (Obi et al. 2016). Amplicons were purified and sequenced at Inqaba Biotechnical Industries (Pty) Ltd South Africa using the genetic analyzer. Sequence chromatograms were manually edited and analyzed using BioEdit and ClustalW software. Sequences were identified based on their closest species using the Basic Local Alignment Search Tool (BLAST) program of the National Centre for Biotechnology Information (NCBI).

The partial 16S rRNA gene sequences in this study are obtainable at the Genbank database under the Accession Numbers MK104459, MK104463, MK104466 and MK104469.

### Kinetic study

The modified Gompertz model was used to evaluate the water hyacinth inoculum potential (Ware and Power 2017; Barua et al. 2019). Application of the model was due to its extensive range of applications in methane production. Measured cumulative methane production was used to evaluate the Gompertz model equation:

1 $$Y=\mathrm{M}\cdot \mathrm{exp}\left\{-\mathrm{exp}\left[\frac{Rm\cdot \mathrm{e}}{\mathrm{M}}\left(\uplambda -\mathrm{t}\right)+1\right]\right\}$$

where Y is the cumulative specific methane production (ml) at time t (days); M represents the maximum methane production (mlCH_4_), *Rm* is the maximum specific rate of methane production (mlCH_4_d^−1^); e is a constant (2.71) while λ represents the lag phase in days. Predicted methane values were plotted against experimental methane values for the determination of a graphic fitting curve. Correlation of the predicted values to the experimental values was established by obtaining the R^2^ value.

### Statistical analysis

Data generated in this study was compared using one-way analysis of variance (ANOVA) to determine significance level at *P* ≤ 0.05. This was to estimate significant differences among the experimental treatments in terms of methane production. A post-hoc test was conducted with Tukey HSD (Honestly significant difference) to identify treatment pairs that differ significantly. Statistical software, SAS version 9.4 statistical software (SAS 1999) was used to conduct the statistical analysis.

## Results

The compositional analysis of water hyacinth (Table 2) shows its elevated moisture and carbohydrate content. The substrate is rich in cellulose and hemicellulose but low in lignin. The existence of macroelements, N, P and K (Table 2) further confirms its potential utilization as a soil ameliorant for plant growth promotion. Analysis of water hyacinth inoculum displayed the reduction of the majority of the outlined properties (Table 2) when compared with fresh water hyacinth.

**Table 2:** Compositional analysis of water hyacinth and water hyacinth inoculum

Properties	Water hyacinth (Quantity)	Water hyacinth inoculum (Quantity)
Dry matter (%)	5.97 ± 0.32	0.31 ± 0.3
Ash (%)	0.96 ± 0.28	0.02 ± 0.11
Protein (%)	1.14 ± 0.45	0.05 ± 0.24
Fat (ether extraction) (%)	0.18 ± 0.29	0.01 ± 0.59
Carbohydrates (%)	3.69 ± 1.13	0.23 ± 1.05
NDF (%)	3.34 ± 0.7	0.22 ± 0.34
ADF (%)	0.68 ± 1.3	0.11 ± 0.87
ADL (%)	0.21 ± 0.7	0.01 ± 0.36
Cellulose (%)	0.47 ± 0.82	^a^
Nitrogen (g/kg)	2.49 ± 1.10	0.348 ± 0.94
Potassium (g/kg)	4.44 ± 0.43	0.491 ± 1.10
Phosphorus (g/kg)	5.02 ± 0.35	0.049 ± 0.2
Carbon/nitrogen (C/N)	14.5	1.6
pH	8.11 ± 0.34	5.14 ± 0.07
Electrical conductivity (mS/m)	1087 ± 1.2	271 ± 0.4

^a^Not available

**Fig. 1:**
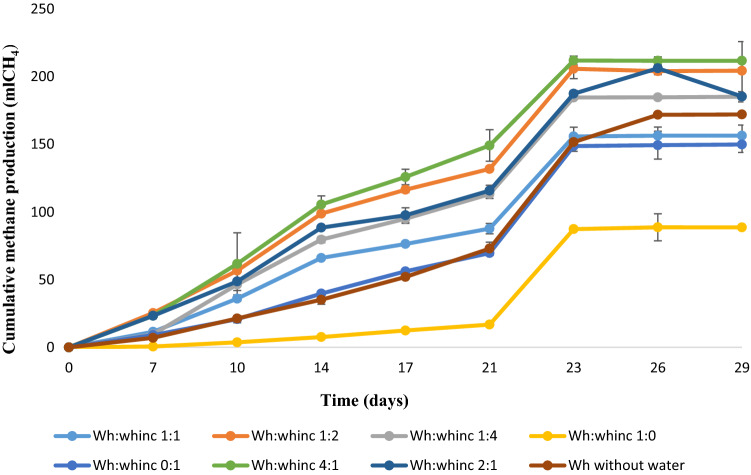
Cumulative methane produced from AD of various ratios of water hyacinth and water hyacinth inoculum. Error bars represent standard deviation (n = 3)

Most treatments with different mixing ratios of Wh and whinc began producing significant methane on the 7th day of AD and methane production increased with time (Fig. 1). All treatments excluding Wh:whinc 2:1 and Wh without water recorded no additional methane after day 23. Treatment without whinc produced the least amount of methane all through the AD period. The ANOVA test for methane production suggested significant variations (*P* < 0.05) among some of the treatments and the post hoc test (Tukeys) revealed that differences existed between treatments Wh:whinc 4:1 and Wh:whinc 1:0; Wh:whinc 1:2 and Wh:whinc 1:0; Wh:whinc 2:1 and Wh:whinc 1:0. However, no significant difference was spotted between treatment Wh:whinc 4:1 and other treatments. Significant differences existed between treatments Wh:whinc 1:0 and other treatments excluding Wh:whinc 0:1 and treatment without water. Treatment Wh:whinc 4:1 portrayed the maximal cumulative methane (0.21 L), next were treatments Wh:whinc 1:2, Wh:whinc 2:1 and Wh:whinc 1:4 with 0.20 L, 0.19 L and 0.19 L of cumulative methane respectively. However, no significant difference existed among the aforementioned treatments with regards to methane production during the batch tests. Biogas composition (CH_4_ and CO_2_) of the different mixing ratios after digestion are reported in the supporting information (Table 6).

Incorporation of water hyacinth inoculum as a co-substrate did not display significant effects on the pH of different mixing ratios both before and after AD (Table 3). The metabolic process of AD was stabilized based on the increased pH of the system and elevated pH values were observed in all the treatments after AD (Table 3). The pH of all mixing ratios ranged between 5.09 and 5.53 before AD and increased up to 8 after AD. The electrical conductivity (EC) values of the resulting digestate as described in Table 4 showed decreased EC values in treatments with more whinc.

**Table 3:** pH values of the treatments before and after digestion

Treatments	Before digestion	After digestion
Wh:whinc 1:1	5.09 ± 0.04^a^	6.16 ± 0.03^a^
Wh:whinc 1:2	5.11 ± 0.03^a^	7.52 ± 0.01^ef^
Wh:whinc 1:4	5.21 ± 0.01^a^	7.15 ± 0.04^b^
Wh:whinc 1:0	5.15 ± 0.04^a^	7.48 ± 0.00^eg^
Wh:whinc 0:1	5.14 ± 0.00^a^	7.76 ± 0.01^c^
Wh:whinc 4:1	5.19 ± 0.03^a^	7.59 ± 0.06^ fg^
Wh:whinc 2:1	5.53 ± 0.04^b^	8.52 ± 0.01^d^

pH values (before digestion and after digestion) with the same letters are not significantly different (P > 0.05)

**Table 4:** Electrical conductivity (EC) of digestates from wh:whinc treatments

Treatments	Electrical conductivity (mS/m)
Wh:whinc 1:1	1750 ± 0.24^a^
Wh:whinc 1:2	232 ± 0.3^b^
Wh:whinc 1:4	243 ± 1.7^b^
Wh:whinc 1:0	1268 ± 0.4^a^
Wh:whinc 0:1	278 ± 0.54^b^
Wh:whinc 4:1	1379 ± 2.7^a^
Wh:whinc 2:1	1832 ± 0.55^a^

EC values with the same letters are not significantly different (P > 0.05)

**Fig. 2:**
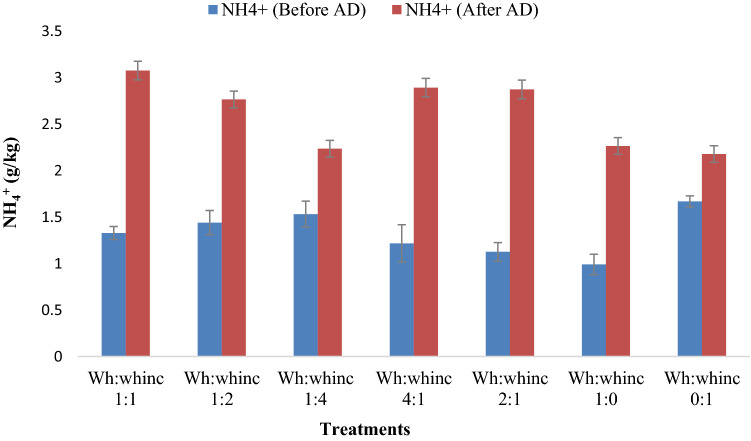
Ammonium content of the treatments before and after digestion. Error bars represent standard deviation (n = 3)

**Fig. 3:**
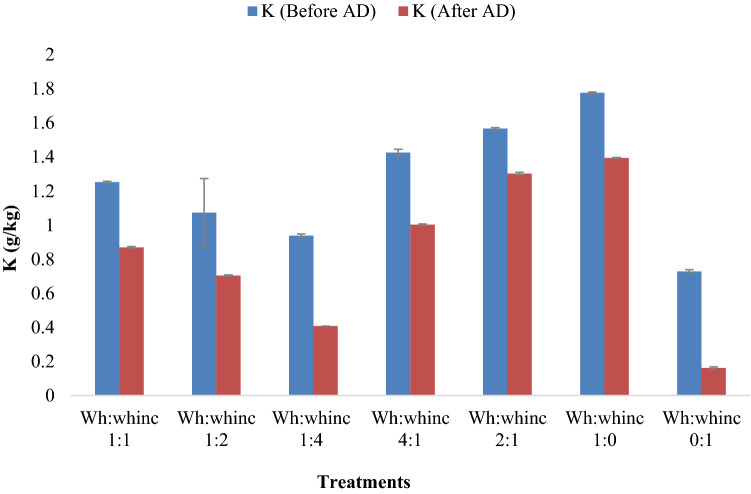
Potassium content of the treatments before and after digestion. Error bars represent standard deviation (n = 3)

**Fig. 4:**
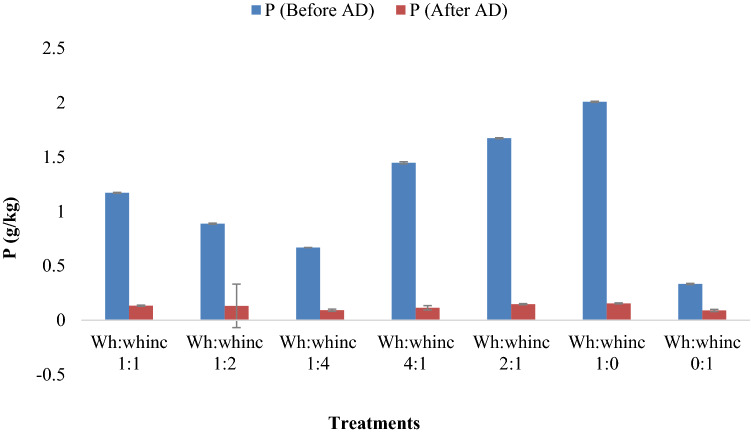
Phosphorus content of the treatments before and after digestion. Error bars represent standard deviation (n = 3)

The result presented on Figs. 2, 3 and 4 all suggested the effect of AD on the macroelements of the anaerobic digesters. Anaerobic digestion improved the nitrogen content of treatments in the form of ammonium as shown in Fig. 2 as significant increase in ammonium concentrations was observed after AD across treatments. Treatments Wh:whinc 1:1, Wh:whinc 4:1, Wh:whinc 2:1 and Wh:whinc 1:0 doubled their ammonium content after AD (Fig. 2). However, the reverse was the case for phosphorus and potassium as their concentrations were reduced after AD (Figs. 3, 4). The phosphorus content of treatment Wh:whinc 1:0 significantly decreased after AD by 85%. Significant differences existed across treatments (*P* < 0.05) before and after AD indicating the effects of AD and ratio variations. Comparison of the mixing ratios with regards to P concentration revealed high levels of P in undigested water hyacinth suggesting the effects of AD on the P content of water hyacinth. A decreasing trend in K content of the treatments after AD was observed (Fig. 3). Higher percentage reduction of K concentration was detected in treatments Wh:whinc 1:4 and Wh:whinc 0:1. Treatments Wh:whinc 1:0 and Wh:whinc 0:1 suggested high content of K in undigested water hyacinth and the impact of AD on K content of water hyacinth.

**Fig. 5:**
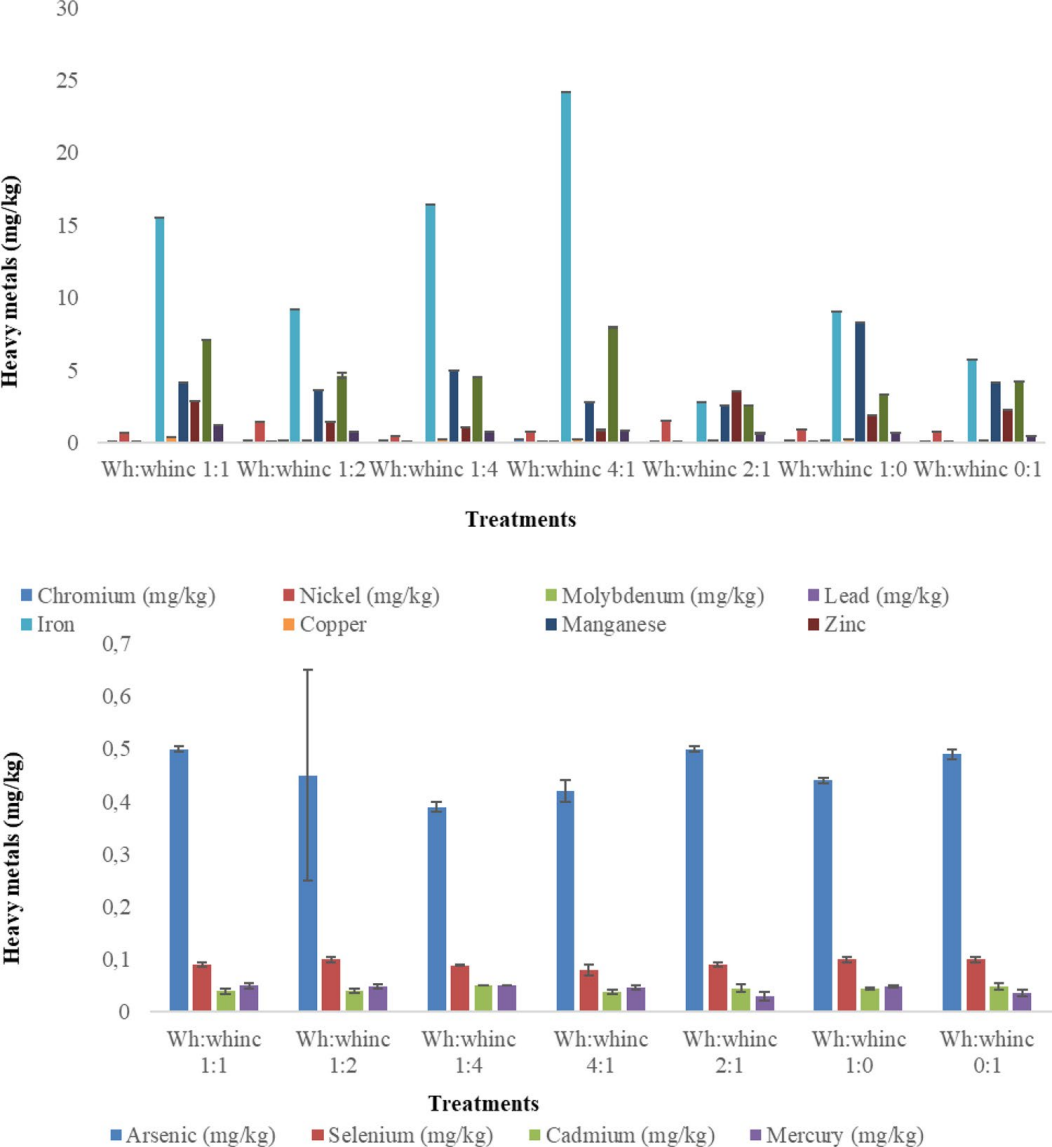
Heavy metals present in the digestate. Error bars represent standard deviation (n = 3)

The concentrations of heavy metals identified in all mixing ratios of the digestate were low as depicted in Fig. 5. The presence of these metals reflects the heavy metal contamination of the aquatic environment (freshwater ecosystem of the Hartbeespoort dam) where the water hyacinth was harvested. The concentration of heavy metals identified in the digestate met the required standard for fertilizers according to the Fertiliser regulations in South Africa (DAFF 2012; Mukhuba et al. 2018). Detection of distinct bands after PCR agarose gel electrophoresis (see Figs. 6, 7 in supporting information) confirmed the amplification of the *nifH* and *phoD* genes with amplicon sizes of 360 bp and 370 bp respectively. Analysis of the 16S rRNA gene sequences identified the organisms as *Pseudomonas stutzeri, Bacillus subtilis, Bacillus pumilus,* and *Bacillus cereus*.

### Kinetic study

Table 5 sums up the outcome of the kinetic study and the Gompertz model fitted the experimental data (Fig. 6). The value of R^2^ measured above 0.95, which showed the potential modelling of methane production. However, R^2^ value has been reported to not essentially specify exact suitability of the experimental data (Ware and Power 2017).

**Table 5:** Kinetic features of water hyacinth inoculum used in this study

Substrate	M (mlCH_4_)	*Rm* (mlCH_4_d^−1^)	λ (days)	R^2^
Water hyacinth inoculum	140	26	2	0.995

## Discussions

Water hyacinth’s suitability as a substrate for biogas production stems from its chemical composition of high moisture and carbohydrate content as well as association of the substrate with microbial entities that are capable of methanogenic activities (Sindhu et al. 2017; Roopnarain et al. 2019). However, some factors could limit methane production during AD of water hyacinth. These factors range from unavailability of essential microorganisms to facilitate the different phases of AD and the lignocellulosic nature of the substrate, to physico-chemical factors which include oxygen content, ammonia content, temperature, pH as well as C/N ratio of the substrate (Rezania et al. 2017; Yang et al. 2019). Efficient AD process has been reported to be in the optimal C/N ratio of 20 – 35 (Bhatt and Tao 2020) and the physico-chemical characterization of water hyacinth used in this study, as depicted in Table 1, shows low C/N ratio, which possibly did not favor methane production. The low bioavailability of the insoluble organic polymeric portions (cellulose and lignin) of the substrate affected hydrolysis as the rate-limiting step of AD of such substrates (Tsapekos et al. 2017). These organic polymers require the actions of extracellular enzymes to be split into simpler components for subsequent metabolism. Secretion of these enzymes is carried out by microorganisms which are mostly obligate anaerobes that are possibly resident in the digesters (Campanaro et al. 2016). The aforementioned suboptimal conditions of the physico-chemical factors possibly affected microbial activities during AD and could have affected methane production. Indigenous microorganisms of water hyacinth inoculum were employed in the metabolic process of methane and soil ameliorant production to minimize the risk of introducing pathogenic microorganisms that could come from various sources of conventional inoculums.

Delayed methane production was observed in some treatments during the course of this study, which could be attributed to prolonged acclimatization, or low concentration of microorganisms such as methanogens, which are essential for methane production. Although the initial concentration of methane was low due to not purging the treatments with nitrogen gas to create anaerobic environment as elevated levels of oxygen may impede the proliferation of methanogens. This challenge only lasted for a short period when the microbes were acclimatizing to the environment, especially the methanogens. Production of methane from the treatments suggests the reduction of the solid fractions, possibly the cellulosic fractions of the substrates. This specifies the metabolic potential of the indigenous microbial entities and their ability to utilize available nutrients/organic matter to generate methane (Hassan et al. 2017). The significant reduction in methane produced from the control treatment (without water hyacinth inoculum) portrays the beneficial effects of water hyacinth inoculum in enhancing methane production as inoculum has been known to host various microorganisms that are favourable to biomethane production (Strang et al. 2017; Rajput and Sheikh 2019). Increased methane production with time in all the treatments shows the relevance of whinc as a co-substrate, treatments without whinc produced the least amount of methane during AD (Fig. 1). Aside from the controls (Wh:whinc 1:0 and Wh:whinc 0:1), treatment Wh:whinc 1:1 produced the least methane and highest ammonia content after AD. This reduced volume of produced methane could be attributed to the inhibitory effect of accumulated ammonia in the digesters (Fig. 2), which probably was due to the mineralization of the abundant nitrogen content of the primary substrate, water hyacinth (Chen et al. 2016; Varanasi et al. 2018). The metabolic process of AD of water hyacinth with whinc enhanced the generation of ammonia from different nitrogen sources in the substrate such as protein, amino acids, urea etc. (Omondi et al. 2019). The presence of large amounts of digestible substrates in treatment Wh:whinc 4:1 prompted the potential production of optimal methane. Utilization of more water hyacinth contributed to increased concentration of mineralised nitrogen, ammonium as treatments Wh:whinc 1:1, Wh:whinc 4:1, Wh:whinc 2:1 and Wh:whinc 1:0 doubled their ammonium content after AD (Fig. 2). The accumulated ammonia is suggested to have limited the growth of potential microbial consortia thereby constraining methane production in some of the treatments (Shi et al. 2017). When compared with the results of previous studies on AD of water hyacinth with dung inoculum, this study recorded a lower methane production (Tasnim et al. 2017). Reports by Westerholm et al. (2018) and Zhang et al. (2022) similarly confirm the detrimental impact of ammonia on synthrophic acetate oxidizing bacteria during AD. Additionally, exclusion of foreign microbes such as those found in cow dung inoculum in the digesters could have also contributed to reduced methane production, due to inefficiency of the indigenous microbial community of water hyacinth inoculum to improve methane production (Horváth et al. 2016).

High moisture content of inoculum has been reported to improve the mixing efficiency of digesters and metabolic activities of indigenous microorganisms, which could enhance the metabolic process of AD (Mir et al. 2016; Muthudineshkumar and Anand 2019). The importance of moisture in AD cannot be overemphasized as a part of this study exhibited the beneficial effect of moisture on methane production. Less methane production was observed in the treatment that was digested without water or water hyacinth inoculum (Fig. 1). Moisture played a vital role in enhancing the dissolution and digestion of the substrate (Guna et al. 2017). Nonetheless, the need to conserve water is imperative as water is fast becoming a scarce commodity attributable to global population growth and changes in climatic conditions (Flörke et al. 2018). Methane is produced by anaerobic methanogens, which are very sensitive to changes in environmental conditions such as pH and temperature. Optimal activity of methanogens in previous studies has been recorded at pH 6.5–7.5 and this supports the results of this study where the pH of most of the treatments (digestate) was in an optimal range that supported the growth of methanogens suggesting stability of the AD process (Rozy et al. 2017; Cerón-Vivas et al. 2019). Increased pH relates to accumulated ammonium, however, the presence of accumulated ammonia in the digesters though toxic to methanogens, further supports the utilization of the resultant digestate as a soil ameliorant (Zhang et al. 2017; Adeleke et al. 2019). Incorporation of whinc during AD promoted the phosphorus content of the digesters considering that treatments without whinc had the least content of P after AD. This study also suggested activation of P solubilization by AD (Liu et al. 2019). Despite the fact that no effect of AD on P solubility was recorded by Bachmann et al. (2016) during AD, low concentrations of phosphorus observed in the digestate in this study could be due to the transformation of phosphorus to various forms of inorganic compounds during AD which could be attributed to the pH of the digesters’ content (Wu et al. 2019, 2021; Li et al. 2020). Such inorganic compounds include struvite, MgNH_4_PO_4_·6H_2_O, hydroxyapatite, Ca_5_(PO_4_)_3_(OH) and vivianite, Fe^++3^(PO_4_)_2_·8(H_2_O). They are known as slow release sources of phosphate to plants; their insolubility decreases their loss during leaching when the digestate is applied as a soil ameliorant (Bachmann et al. 2016; Taşkın et al. 2018). Significant reduction of P and K after AD as observed in Figs. 3 and 4 could also be related to the ability of the high organic content feedstock to provide a favorable environment for the proliferation of microorganisms as the growth of anaerobic microorganisms depends on the availability of macro-nutrients such as P and K, as well as several other inorganic elements that act as micro-nutrients. This conforms to the study of Sawatdeenarunat et al. (2018).

The capacity of water hyacinth to absorb heavy metals and salts in its natural habitat has been related to its phytoremediation abilities (Sidek et al. 2018; Nazir et al. 2020). The stimulatory effect of heavy metals on the metabolic potential of indigenous microorganisms to produce methane has been investigated (Zupančič and Grilc 2012; Romero-Güiza et al. 2016). These metals are beneficial to the microorganisms at certain concentrations and the concentrations of heavy metals identified in the digestate met the required standard for fertilizers according to the Fertiliser regulations in South Africa (DAFF 2012; Mukhuba et al. 2018). This observation further explains the prospective use of the digestate as a soil ameliorant. Heavy metals such as iron, zinc, manganese, copper and nickel which were present in the digestate have been associated with plant growth and productivity while arsenic, chromium, aluminium, cadmium are toxic to plants above selected concentrations (Hassan et al. 2017). The environmental condition of the aquatic ecosystem led to high electrical conductivity (EC) of sampled water hyacinth. The EC level of the substrate is an indication of its salinity and Chen et al. (2020) reported the absolute obstruction of methanogenesis at salinity of > 3000 mS/m, however, the present study reports minimum and maximum EC values of the treatments as 232 mS/m and 1832 mS/m respectively, thus signifying metabolism in all treatments. Digestate resulting from treatments with high concentration of water hyacinth had high EC values (Table 4) and high EC values of digestate treatments which also relates to high ion concentration has been previously linked to high concentration of water hyacinth (Piccoli et al. 2021). This suggests that EC of anaerobic digestate is a function of EC of the substrates prior to AD. Optimal EC levels for some plants ranges from 150 to 250 mS/m and high EC levels have been recorded to interfere with plants ability to absorb nutrients while very low EC could affect productivity (Sharma et al. 2018).

Detection of distinct bands after PCR agarose gel electrophoresis of the *nifH* genes (360 base pairs) as well as the *phoD* genes (370 base pairs) in the digestate samples signifies amplification of genes of interest. Amplification of *phoD* genes in digestate samples signify the presence of phosphate solubilising microorganisms that are capable of producing the enzyme, alkaline phosphatase (Zimmerman et al. 2013; Fraser et al. 2015). The *phoD* gene is one of three homologous genes that encode the enzymes, alkaline phosphatase. These enzyme catalyses the mineralisation of organic phosphate to a form of phosphate (ortophosphate) that is accessible to plants in order to support their growth (Bergkemper et al. 2016; Raimi et al. 2017). The presence of the *nifH* genes indicates the existence of nitrogen fixing microorganisms in the digestate. These organisms are known to convert atmospheric nitrogen gas to plant accessible form of nitrogen (ammonium) through the secretion of nitrogenase enzymes, which are encoded by the *nifH* gene (Zehr and Turner 2001; Gérikas Ribeiro et al. 2018). Consequently, the identification of these genes simply illustrates the viability of the digestate from this study as a potential nitrogen fixing and phosphate solubilising soil ameliorant (Niu et al. 2018).

Bacterial isolates obtained from the water hyacinth inoculum in this study as identified by the 16S rRNA gene sequence analysis characterised them as *Pseudomonas stutzeri, Bacillus cereus, Bacillus subtilis*, and *Bacillus pumilus.* The involvement of these indigenous microbial entities to produce water hyacinth inoculum for biogas and soil ameliorant production was to curtail the risk of pathogenicity of inoculum from other sources such as animal dung. These microorganisms have been previously reported to enhance the degradation of cellulose due to their cellulase producing nature (Siu-Rodas et al. 2018; Dutoit et al. 2019). However, recovery of undigested plant materials at the end of AD period and limited methane production confirms the limited activities of these identified bacteria as well as overall limited bacterial population and diversity in the treatments. Stability of methane production after 29 days of AD cannot be attributed to exhaustion of substrates but limited microbial activities in the metabolism of more recalcitrant components of the organic substrates. Furthermore, these identified bacterial entities (*Pseudomonas stutzeri, Bacillus cereus, Bacillus subtilis*, and *Bacillus pumilus*) have been associated with phosphate solubilisation and nitrogen fixation potential (Mohamed et al. 2018; Saeid et al. 2018; Hashem et al. 2019; Ke et al. 2019). The digestate also possesses readily available plant nutrients that can improve soil fertility and crop productivity, thus maximizing its feasibility as a soil ameliorant (Möller and Müller 2012; Walsh et al. 2012; Sindhu et al. 2017).

In summary, this study is a novel report on the suitability of water hyacinth from the Hartbeespoort dam as an inoculum to enhance methane production. The treatment, Wh:whinc 4:1 presents the ideal mixing ratio for optimal methane production when compared with other treatments. This signifies the requirement of water hyacinth inoculum to enhance the AD process but in low concentrations. The treatment without water hyacinth inoculum (Wh:whinc 1:0) not only exhibited the potential of water hyacinth inoculum to enhance the process of AD of lignocellulosic substrate, it also provided evidence supporting the advantages of utilizing the mixing ratio that resulted in the highest methane output. Although, overall production of low concentration of methane from AD of water hyacinth and water hyacinth inoculum is a function of limited essential microbial diversity and activities, low buffering capacity and accumulation of inhibitory compounds. The study also highlighted the high EC level of water hyacinth from the Hartbeespoort dam; however, the potential of water hyacinth inoculum to contribute to reduced EC levels of the digestate is a benefit to the utilization of the digestate as a soil fertility enhancer. The prospect of improving methane production and the feasibility of the digestate as soil ameliorant via bioaugmentation of the AD process with suitable microbial cultures could be explored.

## Supplementary Information

Below is the link to the electronic supplementary material.

Supplementary file1 (DOCX 183 KB)

## Data Availability

This paper contain data generated or analyzed in the course of this study. Further information relating to data produced in this study can be obtained from the corresponding author on reasonable request.

## References

[CR1] Achinas S, Li Y, Achinas V, Euverink GJW (2019). Biogas potential from the anaerobic digestion of potato peels: process performance and kinetics evaluation. Energies.

[CR2] Adeleke R, Cloete E, Khasa D (2010). Isolation and identification of iron ore-solubilising fungus. S Afr J Sci.

[CR3] Adeleke RA, Nunthkumar B, Roopnarain A, Obi L, Kumar V, Prasad R, Kumar M, Choudhary DK (2019). Applications of plant–microbe interactions in agro-ecosystems. Microbiome in plant health and disease.

[CR4] American Public Health Association (APHA), American Water Works Association (AWWA), Water Environment Federation (WEF) (2017) Standard Methods for the Examination of Water and Wastewater, 23rd edn. APHA-AWWA-WEF, Washington

[CR5] Arutselvy B, Rajeswari G, Jacob S (2021). Sequential valorization strategies for dairy wastewater and water hyacinth to produce fuel and fertilizer. J Food Process Eng.

[CR6] Atta KPT, Maree JP, Onyango MS, Mpenyana-Monyatsi L, Mujuru M (2020). Chemical phosphate removal from Hartbeespoort Dam water South Africa. Water SA.

[CR7] Bachmann S, UptmootEichler-Löbermann RB (2016). Phosphorus distribution and availability in untreated and mechanically separated biogas digestates. Sci Agric.

[CR8] Barua VB, Kalamdhad AS (2019). Biogas production from water hyacinth in a novel anaerobic digester: a continuous study. Process Saf Environ Prot.

[CR9] Barua VB, Rathore V, Kalamdhad AS (2019). Anaerobic co-digestion of water hyacinth and banana peels with and without thermal pretreatment. Renew Energy.

[CR10] Bergkemper F, Kublik S, Lang F, Krüger J, Vestergaar G, Schloter M, Schulz S (2016). Novel oligonucleotide primers reveal a high diversity of microbes which drive phosphorous turnover in soil. J Microbiol Methods.

[CR11] Bhatt AH, Tao L (2020). Economic perspectives of biogas production via anaerobic digestion. Bioengineering.

[CR12] Campanaro S, Treu L, Kougias PG, De Francisci D, Valle G, Angelidaki I (2016). Metagenomic analysis and functional characterization of the biogas microbiome using high throughput shotgun sequencing and a novel binning strategy. Biotechnol Biofuels.

[CR13] Carmo DLD, Lima LBD, Silva CA (2016). Soil fertility and electrical conductivity affected by organic waste rates and nutrient inputs. Rev Bras Cienc Solo.

[CR14] Cerón-Vivas A, Cáceres KT, Rincón A, Cajigas ÁA (2019). Influence of pH and the C/N ratio on the biogas production of wastewater. Rev Fac Ing Univ Antioq.

[CR15] Chen H, Wang W, Xue L, Chen C, Liu G, Zhang R (2016). Effects of ammonia on anaerobic digestion of food waste: process performance and microbial community. Energy Fuels.

[CR16] Chen YT, Yu N, Sun ZY, Gou M, Xia ZY, Tang YQ, Kida K (2020). Acclimation improves methane production from molasses wastewater with high salinity in an upflow anaerobic filter reactor: performance and microbial community dynamics. Appl Biochem Biotechnol.

[CR17] Dennis OE (2015). Effect of inoculums on biogas yield. IOSR J Appl Chem.

[CR18] Dutoit R, Delsaute M, Collet L, Vander Wauven C, Van Elder D, Berlemont R, Richel A, Galleni M, Bauvois C (2019). Crystal structure determination of Pseudomonas stutzeri A1501 endoglucanase Cel5A: the search for a molecular basis for glycosynthesis in GH5_5 enzymes. Acta Crystallogr D.

[CR19] Etta AEB, James E, Ben A, Tiku DR (2017). Biogas generation from co-digestion of four substrates; water hyacinth, cassava peels, poultry droppings and cow dung. Annu Res Rev Biol.

[CR20] Ferraro A, Massini G, Miritana VM, Rosa S, Signorini A, Fabbricino M (2020). A novel enrichment approach for anaerobic digestion of lignocellulosic biomass: process performance enhancement through an inoculum habitat selection. Bioresour Technol.

[CR21] Flörke M, Schneider C, McDonald RI (2018). Water competition between cities and agriculture driven by climate change and urban growth. Nat Sustain.

[CR22] Department of Agriculture, Forestry and Fisheries (2012) Fertilisers, farm feeds, agricultural remedies and stock remedies ACT No. 36 of 1947. Pretoria

[CR23] Fraser TD, Lynch DH, Bent E, Entz MH, Dunfield KE (2015). Soil bacterial phoD gene abundance and expression in response to applied phosphorus and long-term management. Soil Biol Biochem.

[CR24] Fraser TD, Lynch DH, Gaiero J, Khosla K, Dunfield KE (2017). Quantification of bacterial non-specific acid (phoC) and alkaline (phoD) phosphatase genes in bulk and rhizosphere soil from organically managed soybean fields. Appl Soil Ecol.

[CR25] Gérikas Ribeiro C, Lopes dos Santos A, Marie D, Pereira Brandini F, Vaulot D (2018). Small eukaryotic phytoplankton communities in tropical waters off Brazil are dominated by symbioses between Haptophyta and nitrogen-fixing cyanobacteria. ISME J.

[CR26] Guna V, Ilangovan M, Anantha Prasad MG, Reddy N (2017). Water hyacinth: a unique source for sustainable materials and products. ACS Sustain Chem Eng.

[CR27] Hashem A, Tabassum B, Abd Allah EF (2019). *Bacillus subtilis*: a plant-growth promoting rhizobacterium that also impacts biotic stress. Saudi J Biol Sci.

[CR28] Hassan M, Ding W, Umar M, Hei K, Bi J, Shi Z (2017). Methane enhancement and asynchronism minimization through co-digestion of goose manure and NaOH solubilized corn stover with waste activated sludge. Energy.

[CR29] Hindrichsen IK, Kreuzer M, Madsen J, Knudsen KB (2006). Fiber and lignin analysis in concentrate, forage, and feces: detergent versus enzymatic-chemical method. Int J Dairy Sci.

[CR30] Honlah E, Yao Segbefia A, Odame Appiah D, Mensah M, Atakora PO (2019). Effects of water hyacinth invasion on the health of the communities, and the education of children along River Tano and Abby-Tano Lagoon in Ghana. Cogent Soc Sci.

[CR31] Horváth IS, Tabatabaei M, Karimi K, Kumar R (2016). Recent updates on biogas production-a review. Biofuel Res J.

[CR32] Husson O, Brunet A, Babre D, Charpentier H, Durand M, Sarthou JP (2018). Conservation agriculture systems alter the electrical characteristics (Eh, pH and EC) of four soil types in France. Soil Tillage Res.

[CR33] Jones JL, Jenkins RO, Haris PI (2018). Extending the geographic reach of the water hyacinth plant in removal of heavy metals from a temperate Northern Hemisphere river. Sci Rep.

[CR34] Ke X, Feng S, Wang J, Lu W, Zhang W, Chen M, Lin M (2019). Effect of inoculation with nitrogen-fixing bacterium *Pseudomonas stutzeri* A1501 on maize plant growth and the microbiome indigenous to the rhizosphere. Syst Appl Microbiol.

[CR35] Khan AL, Halo BA, Elyassi A, Ali S, Al-Hosni K, Hussain J, Al-Harrasi A, Lee IJ (2016). Indole acetic acid and ACC deaminase from endophytic bacteria improves the growth of *Solanum lycopersicum*. Electron J Biotechnol.

[CR36] Kumar B, Bhardwaj N, Agrawal K, Chaturvedi V, Verma P (2020). Current perspective on pretreatment technologies using lignocellulosic biomass: an emerging biorefinery concept. Fuel Process Technol.

[CR37] Kunatsa T, Zhang L, Xia X (2020). Biogas potential determination and production optimisation through optimal substrate ratio feeding in co-digestion of water hyacinth, municipal solid waste and cow dung. Biofuels.

[CR38] Li Y, Jing Y, Zhang Z, Jiang D, Zhang Q, Hu J, Zhang H, He C, Zhu S (2020). Kinetics of methane production from the co-digestion of cow dung, pig manure and corn straw. J Biobased Mater.

[CR39] Lianhua L, Feng Z, Yongming S, Zhenhong Y, Xiaoying K, Xianyou Z, Hongzhi N (2014). Low-cost additive improved silage quality and anaerobic digestion performance of napiergrass. Bioresour Technol.

[CR40] Lin R, Cheng J, Song W, Ding L, Xie B, Zhou J, Cen K (2015). Characterisation of water hyacinth with microwave-heated alkali pretreatment for enhanced enzymatic digestibility and hydrogen/methane fermentation. Bioresour Technol.

[CR41] Liu YH, Huang CJ, Chen CY (2010). Identification and transcriptional analysis of genes involved in *Bacillus cereus*-induced systemic resistance in Lilium. Biol Plant.

[CR42] Liu J, Deng S, Qiu B, Shang Y, Tian J, Bashir A, Cheng X (2019). Comparison of pretreatment methods for phosphorus release from waste activated sludge. Chem Eng J.

[CR43] Martínez-Gutiérrez E (2018). Biogas production from different lignocellulosic biomass sources: advances and perspectives. 3 Biotech.

[CR44] Mir MA, Hussain A, Verma C (2016). Design considerations and operational performance of anaerobic digester: a review. Cogent Eng.

[CR45] Mohamed EA, Farag AG, Youssef SA (2018). Phosphate solubilization by *Bacillus subtilis* and *Serratia marcescens* isolated from tomato plant rhizosphere. J Environ Prot.

[CR46] Möller K, Müller T (2012). Effects of anaerobic digestion on digestate nutrient availability and crop growth: a review. Eng Life Sci.

[CR47] Mudhoo A, Kumar S (2013). Effects of heavy metals as stress factors on anaerobic digestion processes and biogas production from biomass. Int J Sci Environ Technol.

[CR48] Mukhongo RW, Tumuhairwe JB, Ebanyat P, AbdelGadir AH, Thuita M, Masso C (2017). Combined application of biofertilizers and inorganic nutrients improves sweet potato yields. Front Plant Sci.

[CR49] Mukhuba M, Roopnarain A, Adeleke R, Moeletsi M, Makofane R (2018). Comparative assessment of bio-fertiliser quality of cow dung and anaerobic digestion effluent. Cogent Food Agric.

[CR50] Muthudineshkumar R, Anand R (2019). Anaerobic digestion of various feedstocks for second-generation biofuel production. Azad K(ed) Advances in eco-fuels for a sustainable environment.

[CR51] Nazir MI, Idrees I, Idrees P, Ahmad S, Ali Q, Malik A (2020). Potential of water hyacinth (*Eichhornia crassipes *L.) for phytoremediation of heavy metals from waste water. Biol Clin Sci Res J.

[CR52] Niu X, Song L, Xiao Y, Ge W (2018). Drought-tolerant plant growth-promoting rhizobacteria associated with foxtail millet in a semi-arid agroecosystem and their potential in alleviating drought stress. Front Microbiol.

[CR53] Njogu P, Kinyua R, Muthoni P, Nemoto Y (2021). Biogas production using water hyacinth (*Eicchornia crassipes*) for electricity generation in Kenya. Energy Power Eng.

[CR54] Nkuna R, Roopnarain A, Adeleke R (2019). Effects of organic loading rates on microbial communities and biogas production from water hyacinth: a case of mono-and co-digestion. J Chem Technol Biotechnol.

[CR55] Obi LU, Atagana HI, Adeleke RA (2016). Isolation and characterisation of crude oil sludge degrading bacteria. Springerplus.

[CR56] Obi L, Atagana H, Adeleke R, Maila M, Bamuza-Pemu E (2020). Potential microbial drivers of biodegradation of polycyclic aromatic hydrocarbons in crude oil sludge using a composting technique. J Chem Technol Biotechnol.

[CR57] Omondi EA, Ndiba PK, Njuru PG (2019). Characterization of water hyacinth (*E. crassipes*) from Lake Victoria and ruminal slaughterhouse waste as co-substrates in biogas production. SN Appl Sci.

[CR58] Panuccio MR, Attinà E, Basile C, Mallamaci C, Muscolo A (2016). Use of recalcitrant agriculture wastes to produce biogas and feasible biofertilizer. Waste Biomass Valoriz.

[CR59] Peng H, Wang Y, Tan TL, Chen Z (2020). Exploring the phytoremediation potential of water hyacinth by FTIR Spectroscopy and ICP-OES for treatment of heavy metal contaminated water. Int J Phytoremediat.

[CR60] Piccoli I, Virga G, Maucieri C, Borin M (2021). Digestate liquid fraction treatment with filters filled with recovery materials. Water.

[CR61] Qin S, Zhang YJ, Yuan B, Xu PY, Xing K, Wang J, Jiang JH (2014). Isolation of ACC deaminase-producing habitat-adapted symbiotic bacteria associated with halophyte *Limonium sinense* (Girard) Kuntze and evaluating their plant growth-promoting activity under salt stress. Plant Soil.

[CR62] Raimi A, Adeleke R, Roopnarain A (2017). Soil fertility challenges and Biofertiliser as a viable alternative for increasing smallholder farmer crop productivity in sub-Saharan Africa. Cogent Food Agric.

[CR63] Rajput AA, Sheikh Z (2019). Effect of inoculum type and organic loading on biogas production of sunflower meal and wheat straw. Sustain Environ Res.

[CR64] Ramirez A, Pérez S, Flórez E, Acelas N (2021). Utilization of water hyacinth (*Eichhornia crassipes*) rejects as phosphate-rich fertilizer. J Environ Chem Eng.

[CR65] Rezania S, Din MFM, Taib SM, Sohaili J, Chelliapan S, Kamyab H, Saha BB (2017). Review on fermentative biohydrogen production from water hyacinth, wheat straw and rice straw with focus on recent perspectives. Int J Hydrog Energy.

[CR66] Risberg K, Cederlund H, Pell M, Arthurson V, Schnürer A (2017). Comparative characterization of digestate versus pig slurry and cow manure—chemical composition and effects on soil microbial activity. Waste Manag.

[CR67] Romero-Güiza MS, Vila J, Mata-Alvarez J, Chimenos JM, Astals S (2016). The role of additives on anaerobic digestion: a review. Renew Sust Energ Rev.

[CR68] Roopnarain A, Mukhuba M, Adeleke R, Moeletsi M (2017). Biases during DNA extraction affect bacterial and archaeal community profile of anaerobic digestion samples. 3 Biotech.

[CR69] Roopnarain A, Nkuna R, Ndaba B, Adeleke R (2019). New insights into the metagenomic link between pre-treatment method, addition of an inoculum and biomethane yield during anaerobic digestion of water hyacinth (*Eichhornia crassipes*). J Chem Technol Biotechnol.

[CR70] Rozy R, Dar RA, Phutela UG (2017). Optimization of biogas production from water hyacinth (*Eichhornia crassipes*). J Appl Nat Sci.

[CR71] Saeid A, Prochownik E, Dobrowolska-Iwanek J (2018). Phosphorus solubilization by Bacillus species. Molecules.

[CR72] Safauldeen SH, Abu Hasan H, Abdullah SRS (2019). Phytoremediation efficiency of water hyacinth for batik textile effluent treatment. J Ecol Eng.

[CR73] Saidu M, Yuzir A, Salim MR, Azman S, Abdullah N (2014). Biological pre-treated oil palm mesocarp fibre with cattle manure for biogas production by anaerobic digestion during acclimatization phase. Int Biodeterior Biodegrad.

[CR74] Sakurai M, Wasaki J, Tomizawa Y, Shinano T, Osaki M (2008). Analysis of bacterial communities on alkaline phosphatase genes in soil supplied with organic matter. Soil Sci Plant Nutr.

[CR75] Sarto S, Hildayati R, Syaichurrozi I (2019). Effect of chemical pretreatment using sulfuric acid on biogas production from water hyacinth and kinetics. Renew Energy.

[CR76] SAS Institute, Inc. (1999) SAS/STAT User's Guide, Version 9, 1st printing, vol 2. SAS Institute Inc, SAS Campus Drive, Cary, North Carolina 27513

[CR77] Sawatdeenarunat C, Nam H, Adhikari S, Sung S, Khanal SK (2018). Decentralized biorefinery for lignocellulosic biomass: integrating anaerobic digestion with thermochemical conversion. Bioresour Technol.

[CR78] Schroyen M, Vervaeren H, Van Hulle SW, Raes K (2014). Impact of enzymatic pretreatment on corn stover degradation and biogas production. Bioresour Technol.

[CR79] Sharma N, Acharya S, Kumar K, Singh N, Chaurasia OP (2018). Hydroponics as an advanced technique for vegetable production: an overview. J Soil Water Conserv.

[CR80] Shen S, Nges IA, Yun J, Liu J (2014). Pre-treatments for enhanced biochemical methane potential of bamboo waste. Chem Eng J.

[CR81] Shenoy A, Bansal V, Shukla BK (2022). Treatability of effluent from small scale dye shop using water hyacinth. Mater Today Proc.

[CR82] Shi X, Lin J, Zuo J, Li P, Li X, Guo X (2017). Effects of free ammonia on volatile fatty acid accumulation and process performance in the anaerobic digestion of two typical bio-wastes. J Environ Sci.

[CR83] Sidek NM, Abdullah SRS, Draman SFS, Rosli MMM, Sanusi MF (2018). Phytoremediation of abandoned mining lake by water hyacinth and water lettuces in constructed wetlands. J Teknol.

[CR84] Sindhu R, Binod P, Pande A, Madhavan A, Alphonsa JA, Vivek N, Gnansounou E, Castro E, Faraco V (2017). Water hyacinth a potential source for value addition: an overview. Bioresour Technol.

[CR85] Siu-Rodas Y, de lost Angeles Calixto-Romo M, Guillén-Navarro K, Sánchez JE, Zamora-Briseno JA, Amaya-Delgado L (2018). Bacillus subtilis with endocellulase and exocellulase activities isolated in the thermophilic phase from composting with coffee residues. Rev Argent Microbiol.

[CR86] Souza RD, Ambrosini A, Passaglia LM (2015). Plant growth-promoting bacteria as inoculants in agricultural soils. Genet Mol Biol.

[CR87] Strang O, Ács N, Wirth R, Maróti G, Bagi Z, Rákhely G, Kovács KL (2017). Bioaugmentation of the thermophilic anaerobic biodegradation of cellulose and corn stover. Anaerobe.

[CR88] Taşkın MB, Şahin Ö, Taskin H, Atakol O, Inal A, Gunes A (2018). Effect of synthetic nano-hydroxyapatite as an alternative phosphorus source on growth and phosphorus nutrition of lettuce (*Lactuca sativa* L.) plant. J Plant Nutr.

[CR89] Tasnim F, Iqbal SA, Chowdhury AR (2017). Biogas production from anaerobic co-digestion of cow manure with kitchen waste and Water Hyacinth. Renew Energy.

[CR90] Tsapekos P, Kougias PG, Vasileiou SA, Treu L, Campanaro S, Lyberatos G, Angelidaki I (2017). Bioaugmentation with hydrolytic microbes to improve the anaerobic biodegradability of lignocellulosic agricultural residues. Bioresour Technol.

[CR91] Unpaprom Y, Pimpimol T, Whangchai K, Ramaraj R (2021). Sustainability assessment of water hyacinth with swine dung for biogas production, methane enhancement, and biofertilizer. Biomass Convers Biorefin.

[CR92] Van Soest PV, Robertson JB, Lewis B (1991). Methods for dietary fiber, neutral detergent fiber, and nonstarch polysaccharides in relation to animal nutrition. Int J Dairy Sci.

[CR93] Varanasi JL, Kumari S, Das D (2018). Improvement of energy recovery from water hyacinth by using integrated system. Int J Hydrog Energy.

[CR94] Walsh JJ, Jones DL, Edwards-Jones G, Williams AP (2012). Replacing inorganic fertilizer with anaerobic digestate may maintain agricultural productivity at less environmental cost. J Plant Nutr Soil Sci.

[CR95] Ware A, Power N (2017). Modelling methane production kinetics of complex poultry slaughterhouse wastes using sigmoidal growth functions. Renew Energy.

[CR96] Westerholm M, Müller B, Singh A, Karlsson Lindsjö O, Schnürer A (2018). Detection of novel syntrophic acetate-oxidizing bacteria from biogas processes by continuous acetate enrichment approaches. Microb Biotechnol.

[CR97] Wu Y, Luo J, Zhang Q, Aleem M, Fang F, Xue Z, Cao J (2019). Potentials and challenges of phosphorus recovery as vivianite from wastewater: a review. Chemosphere.

[CR98] Wu M, Liu J, Gao B, Sillanpää M (2021). Phosphate substances transformation and vivianite formation in P-Fe containing sludge during the transition process of aerobic and anaerobic conditions. Bioresour Technol.

[CR99] Yang Z, Wang W, Liu C, Zhang R, Liu G (2019). Mitigation of ammonia inhibition through bioaugmentation with different microorganisms during anaerobic digestion: selection of strains and reactor performance evaluation. Water Res.

[CR100] Yu L, Zhang W, Liu H, Wang G, Liu H (2018). Evaluation of volatile fatty acids production and dewaterability of waste activated sludge with different thermo-chemical pretreatments. Int Biodeterior Biodegrad.

[CR101] Zehr JP, Turner PJ (2001). Nitrogen fixation: nitrogenase genes and gene expression. Methods Microbiol.

[CR102] Zhang M, Lin Q, Rui J, Li J, Li X (2017). Ammonium inhibition through the decoupling of acidification process and methanogenesis in anaerobic digester revealed by high throughput sequencing. Biotechnol Lett.

[CR103] Zhang H, Yuan W, Dong Q, Wu D, Yang P, Peng Y, Li L, Peng X (2022). Integrated multi-omics analyses reveal the key microbial phylotypes affecting anaerobic digestion performance under ammonia stress. Water Res.

[CR104] Zimmerman AE, Martiny AC, Allison SD (2013). Microdiversity of extracellular enzyme genes among sequenced prokaryotic genomes. ISME J.

[CR105] Zupančič GD, Grilc V, Kumar S (2012). Anaerobic treatment and biogas production from organic waste. Management of organic waste.

