# Supplementary material for: Bioaugmentation potential of inoculum derived from anaerobic digestion feedstock for enhanced methane production using water hyacinth
Source: World J Microbiol Biotechnol. 2023 Apr 10;39(6):153. doi: 10.1007/s11274-023-03600-9 (PMC10083160; doi:10.1007/s11274-023-03600-9)
Supplement: Supplementary file 1 — Supplementary file1 (DOCX 183 KB) [file 11274_2023_3600_MOESM1_ESM.docx]

**Supplementary data**

**Table 6: B**iogas composition (after AD) of the different mixing ratios of Wh:whinc used in this study

| **Treatments** | **CH_4_ (L)** | **CO_2_ (L)** |
| --- | --- | --- |
| Wh:whinc 1:1 | 0.156 | 0.143 |
| Wh:whinc 1:2 | 0.204 | 0.167 |
| Wh:whinc 1:4 | 0.211 | 0.142 |
| Wh:whinc 1:0 | 0.089 | 0.166 |
| Wh:whinc 0:1 | 0.149 | 0.144 |
| Wh:whinc 4:1 | 0.211 | 0.222 |
| Wh:whinc 2:1 | 0.185 | 0.186 |
|  |  |  |


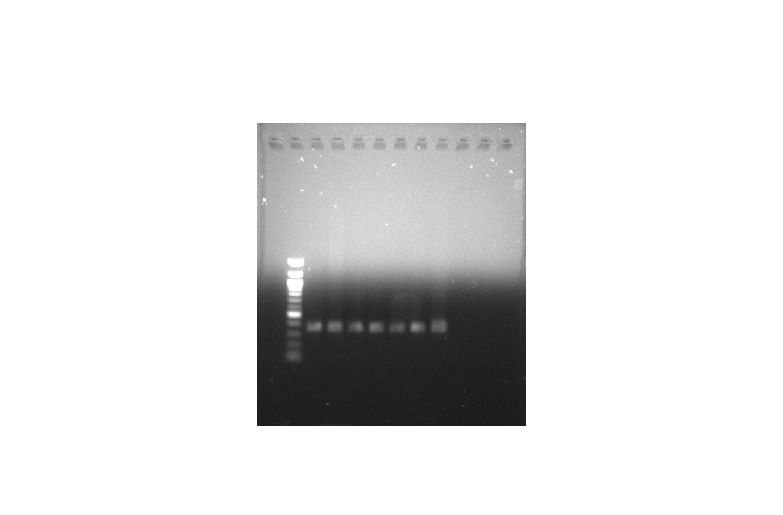


**L 1 2 3 4 5 6 7**

360bp

**Fig. 6**: Agarose gel electrophoresis (1%) of PCR amplified *nifH* genes using specific primers. Lane L is the 100bp DNA size marker. Lanes 1, 2, 3, 4, 5, 6 & 7 represent the seven basic treatments examined in this study.


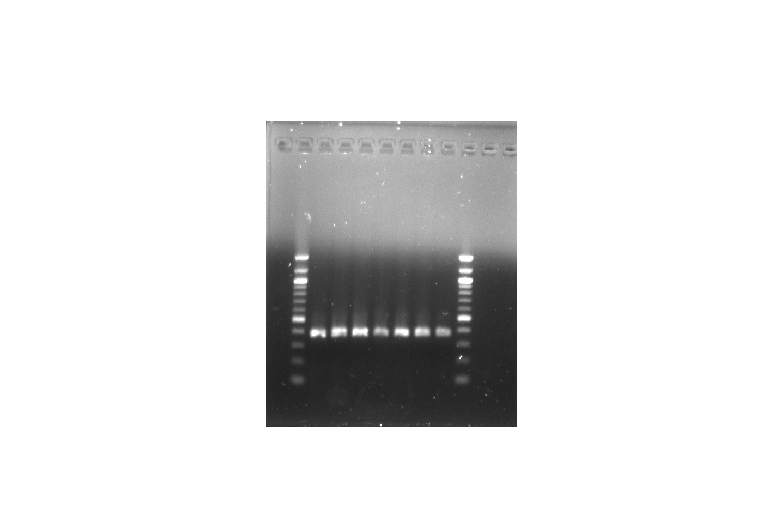


**L 1 2 3 4 5 6 7 L**

370bp

**Fig. 7**: Agarose gel electrophoresis (1%) of PCR amplified *phoD* genes using specific primers. Lanes L are the 100bp DNA size marker. Lanes 1, 2, 3, 4, 5, 6 & 7 represent the seven basic treatments examined in this study.
